# Case Report: Steroids for diabetic myonecrosis in ESKD: an unconventional treatment with unexpected success

**DOI:** 10.3389/fneph.2025.1618775

**Published:** 2025-09-02

**Authors:** Justin David Tse, Sristhi Laller, Sourabh Kharait

**Affiliations:** ^1^ Department of Graduate Medical Education, Internal Medicine Residency, Sutter Roseville Medical Center, Roseville, CA, United States; ^2^ Summit Nephrology Medical Group, Inc., Sutter Roseville Medical Center, Roseville, CA, United States

**Keywords:** myonecrosis, end stage kidney disease, type 1 diabetes, diabetic nephropathy, cellulitis, myositis

## Abstract

**Introduction:**

Myonecrosis is a rare but serious complication of diabetes, particularly in patients with end-stage kidney disease (ESKD), characterized by ischemic necrosis of the skeletal muscles. Its diagnosis is often delayed due to overlapping presentations with cellulitis or deep vein thrombosis. Treatment is traditionally limited to supportive measures such as rest and pain control, which remains the cornerstone. The role of corticosteroids remains controversial in this condition as its effectiveness and utility are not widely understood. This case highlights the unconventional use of corticosteroids in the management of refractory diabetic myonecrosis, emphasizing their potential in mitigating inflammation and promoting recovery.

**Case report:**

We present a 31-year-old woman with ESKD on hemodialysis and a history of type 1 diabetes who presented with recurrent, debilitating pain and swelling in the right lower extremity. Despite a comprehensive workup, including MRI and a muscle biopsy confirming myonecrosis, the patient’s symptoms persisted despite conventional supportive care. Following a multidisciplinary discussion, corticosteroid therapy was initiated, resulting in dramatic symptom resolution within 48 h. The patient experienced significant pain reduction, improved mobility, and decreased swelling, allowing for discharge on a tapered steroid regimen. Notably, a subsequent recurrence of myonecrosis in a different muscle group also responded favorably to corticosteroid treatment, further underscoring its therapeutic potential in the management of patients with this condition.

**Discussion/conclusion:**

This case underscores the importance of considering corticosteroids as an adjunctive therapy in refractory diabetic myonecrosis, particularly in patients who fail to respond to standard care. A detailed workup, a high degree of suspicion, distinct clinical findings, and imaging such as MRI, along with muscle biopsy, can accurately diagnose this condition. While corticosteroids are not routinely used due to their potential risks, their dramatic effect in this patient highlights the need for further research to better understand their role and to refine treatment strategies. By expanding the therapeutic approach to diabetic myonecrosis, this case provides valuable insights for improving outcomes in this rare and challenging condition. This case opens the door for the exploration of corticosteroids as an adjunctive therapy in similar diabetic patients with ESKD and refractory myonecrosis.

## Introduction

Myonecrosis, or muscle infarction, is a rare but serious complication, particularly in patients undergoing dialysis for end-stage renal disease (end-stage kidney disease, ESKD). The diagnosis of myonecrosis is specifically challenging, given its clinical presentation being similar to cellulitis or deep vein thrombosis (DVT) (1–3). Diabetic myonecrosis is exceedingly rare in patients with ESKD, with limited reported cases, likely due to its frequent misdiagnosis as cellulitis or DVT. While the pathophysiology of myonecrosis often relates to microvascular compromise and metabolic disturbances, its presentation in ESKD patients without severe glycemic derangements poses an additional diagnostic and therapeutic challenge. Typically, supportive care with rest and pain control is the cornerstone of treatment, and the role of steroids remains debated, primarily reserved for those with an inflammatory component such as active autoimmune myositis (1–5). This case report sheds light on a rare and atypical scenario where steroid therapy yielded a remarkable improvement in a patient with ESKD and myonecrosis who presented with recurrent symptoms of muscle pain that were unresponsive to conventional supportive care. As many patients with myonecrosis present with “myositis-like” symptoms with an elevation of the inflammatory markers, this case highlights the possible selective role of short-term steroid use in an individual with well-controlled diabetes who was otherwise non-responsive to supportive care.

## Case report

We present the case of a 31-year-old woman with a past medical history of type 1 diabetes, retinopathy of the right eye, ESKD on hemodialysis, hypertension, and previous gastric ulcers with gastrointestinal (GI) bleeding secondary to *Helicobacter pylori* who presented to the emergency department with complaints of right lower extremity swelling and pain initially concerning for cellulitis *vs*. DVT. Pain was categorized below the knee and up to the foot on the right side, which was associated with numbness and tingling. She denied any proceeding trauma or wounds with no associated systemic symptoms such as fever, chills, shortness of breath, or night sweats. Cardiovascular and pulmonary review of systems was negative for chest pain or shortness of breath. The patient denied a history of any insect bite or recent travel with prolonged immobilization. She reported compliance with her dialysis treatments and usually met her estimated dry weight. Her vital signs were stable, and she did not demonstrate any concerns for sepsis. Laboratories were significant for a serum sodium level of 128 mmol/L, as well as a glucose level of 418 mg/dl, with elevated blood urea nitrogen and serum creatinine, as expected for a patient on hemodialysis. Additional laboratory tests indicated an elevation of inflammatory biomarkers, with an erythrocyte sedimentation rate (ESR) of 106 mm/h and a C-reactive protein (CRP) of 30.3 mg/L.

**Figure 1:**
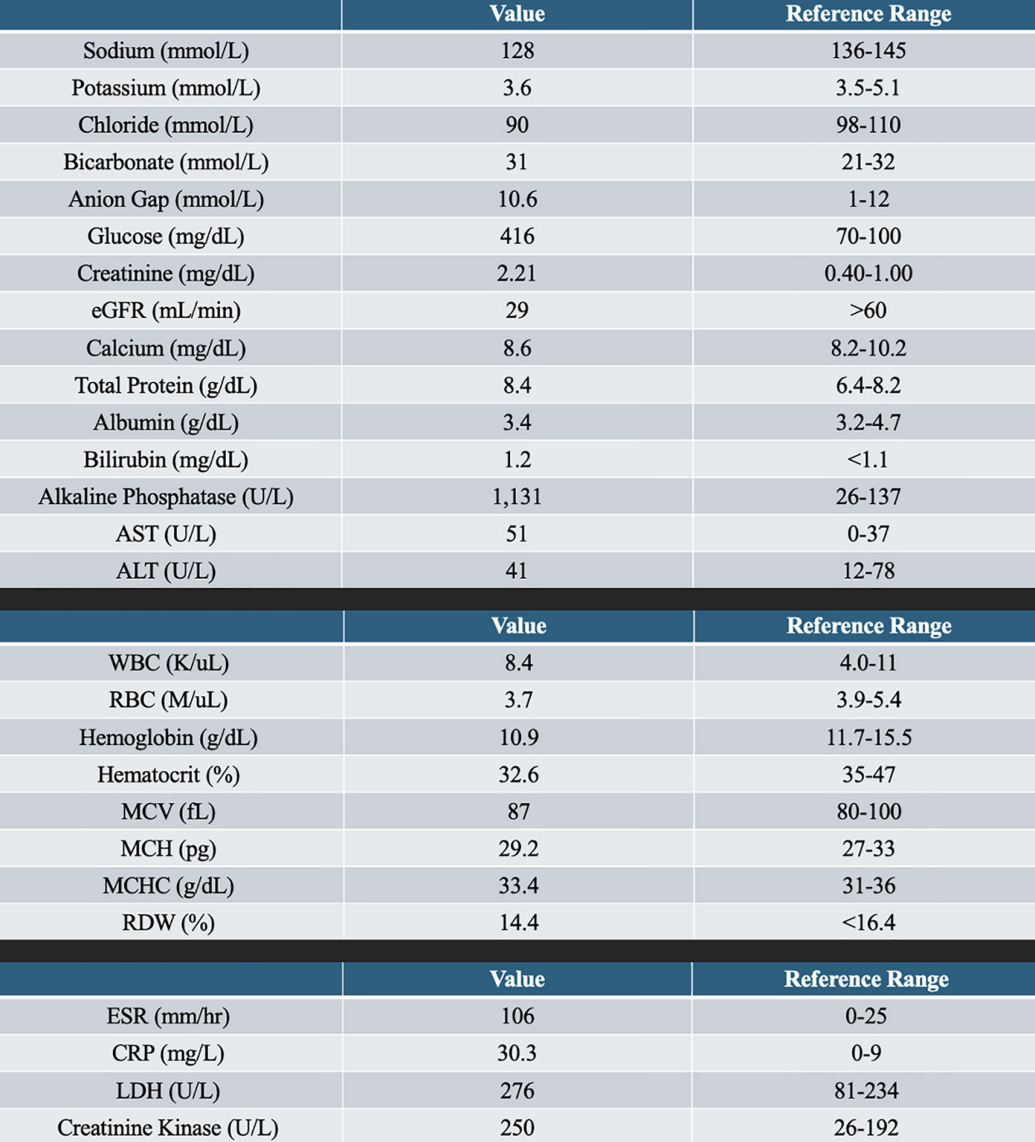
Complete metabolic panel, complete blood count, and other serological markers.

Her muscle injury biomarkers were mildly elevated [aspartate aminotransferase (AST) = 51 U/L, lactate dehydrogenase (LDH) = 276 U/L, and creatine kinase (CK) = 250 U/L], which, although above the normal range, were significantly lower than that typically observed in classical polymyositis, dermatomyositis, or rhabdomyolysis, where CK often reaches levels in the thousands or higher. Specifically, the creatine kinase (CK) level was 250 U/L, the LDH was 276 U/L, and the serum alkaline phosphatase was elevated at 1,131 U/L, indicating either muscle or bone mineral disease. Glycemic control was decent, with A1C of 7.3. See **Figure 1** for the remaining laboratory work.

Physical exam was notable for right lower extremity swelling of the calf and foot, with significant tenderness to palpation. In addition, she had considerable tenderness around her right calf. The temperature of her extremities was normal, and there was no erythema, making the diagnosis of cellulitis essentially void. Pulses were palpable, with intact sensation, but with subjective experiences of tingling. Given concerns for necrotizing fasciitis *vs*. occult cellulitis with micro-abscesses, a stat CT of the lower extremity was obtained, orthopedic surgery was consulted, and broad-spectrum antibiotics, including vancomycin and clindamycin, were empirically started after the blood cultures were sent. During this time, the patient provided more history of similar presentations at least twice that required hospitalization at a nearby medical center. She also notified the staff that, during her last hospitalization, she underwent a muscle biopsy for diagnosis, the results of which were not available for immediate review. A lower extremity duplex ultrasound ruled out DVT in either leg.

**Figure 2:**
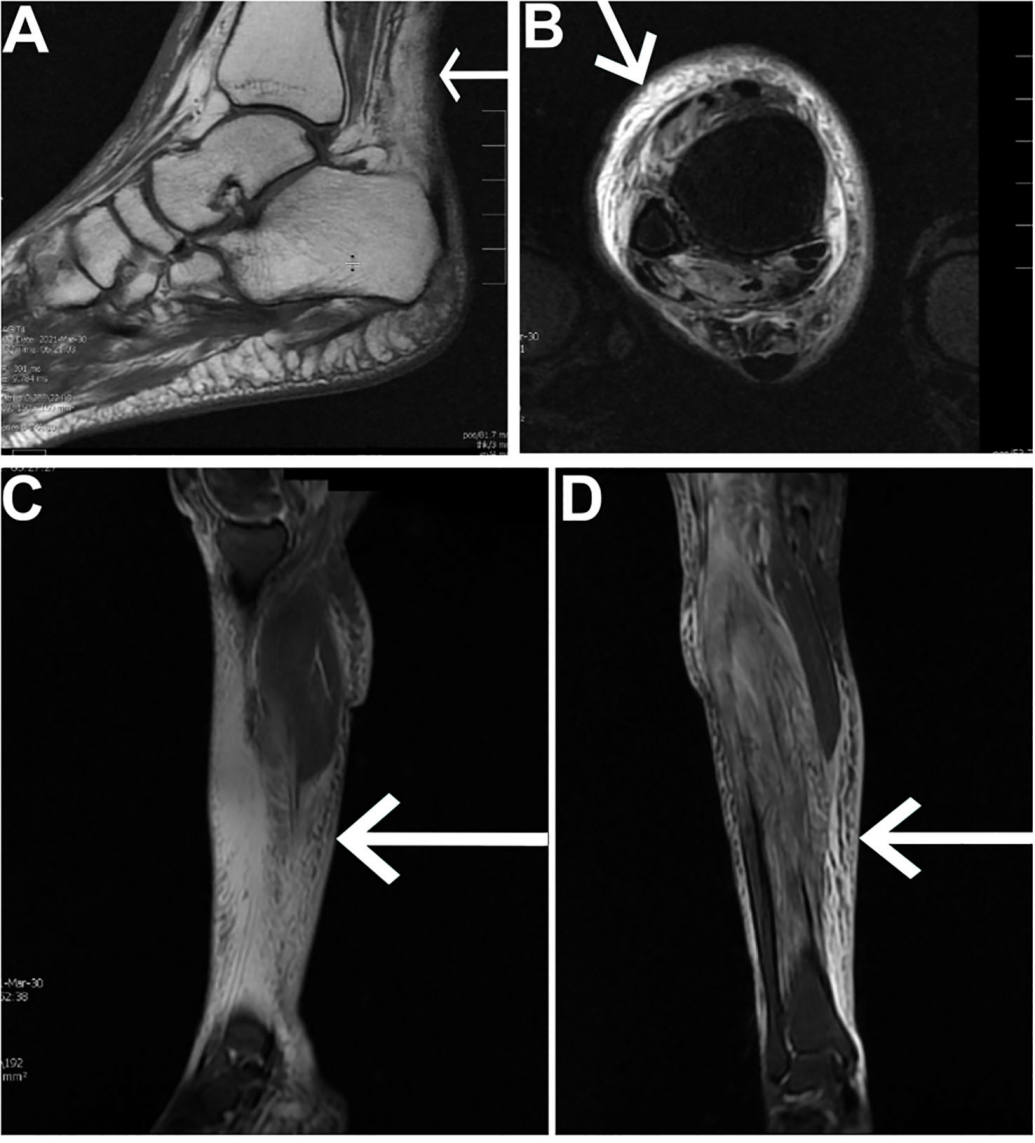
MRI of the right lower extremity. **(A)** T1-weighted sagittal view of the right ankle and foot demonstrating diffuse muscle and subcutaneous edema. **(B)** T2-weighted/short-tau inversion recovery (STIR) axial view of the right lower extremity at the level of the ankle demonstrating diffuse inflammation. **(C)** T2-weighted/STIR sagittal view of the right lower extremity demonstrating diffuse inflammation of the calf, most severe along the anterior–medial aspect. **(D)** T2-weighted/STIR coronal view of the right lower extremity demonstrating diffuse inflammation.

After pain control, a CT of the right lower extremity was completed, which demonstrated diffuse subcutaneous edema extending from the right knee through the right ankle, most consistent with cellulitis or myonecrosis. Given the discrepancy in the clinical findings (i.e., lack of fever or erythema and negative duplex of the lower extremities), an MRI was requested to rule out a diagnosis of myonecrosis and for confirmation. The MRI confirmed diffuse edema of the muscles and the subcutaneous tissue throughout the right ankle, calf, and proximal foot. No abscesses or fluid collections were seen (**Figure 2**). Given no concerns of necrotizing fasciitis or cellulitis, abscesses, and the negative blood cultures, antibiotics were discontinued with a tentative diagnosis of myonecrosis. A tagged white blood cell scan and a nuclear medicine scan were also completed to uncover any occult infection, which demonstrated an asymmetric uptake at the marrow of the right distal femur and proximal tibia, which was deemed to be reactive in nature. A muscle biopsy report was obtained after 48 h, which demonstrated findings suggestive of myonecrosis characterized by patterns of muscle injury, necrosis of focal myofibers, and edema without significant inflammatory infiltration in the muscle tissue. Prior records indicated long‑standing glycemic variability with intermittent hyperglycemia; her A1c on presentation was 7.3%.

Given the unusual presentation in a young woman, an autoimmune workup was completed, which demonstrated no signs of inflammatory myositis. Specifically, the aldolase level was 7.2 U/L, which was within normal limits, and the alkaline phosphatase isoenzyme level was 772 U/L. The antinuclear antibodies (ANA), anti-Jo 1 antibodies, complement levels, and rheumatoid factor were within normal limits. During this time, the patient continued to have severe pain clinically despite supportive care and was unable to walk or stand for more than a few minutes. As the infectious workup was negative, a trial of prednisone was reasonable after deliberation and the relative contraindication of non-steroidal anti-inflammatory drugs (NSAIDs). The patient was started on a 60-mg daily dose of prednisone along with supportive treatment with rest, ice, and pain management. Within 48 h, the patient significantly improved, with a reduction in the need for pain medications, improved mobility, and weight transfer. Prior to steroid therapy, the patient’s pain was rated 9/10 and she had severely limited mobility and daily activities. Within 48 h post-steroids, the pain reduced to 2/10, enabling independent ambulation. Blood glucose was monitored every 4–6 h during steroid treatment, and the insulin dosages were adjusted accordingly. Edema of the right lower extremity, as well as the tenderness, dramatically improved on day 3. By the time of discharge, the patient’s inflammatory markers (i.e., ESR and CRP) had significantly decreased, mirroring the resolution of inflammation and correlating with her clinical improvement post-steroid therapy. While she required adjustment in her insulin dosage due to hyperglycemia, which was expected due to the steroid dosing, her overall condition improved. She was discharged on an initial prednisone dose of 60 mg daily, tapered gradually over a 3-month period.

A few weeks later, the patient returned to the emergency department with similar symptoms, but with worsening upper left arm pain. CT of the upper extremities demonstrated subcutaneous edema along the proximal left forearm; however, no abscess was suspected. Similar to her last admission, she was started on antibiotics for suspected cellulitis *vs*. an arteriovenous fistula (AVF) infection, but which was quickly discontinued given the negative blood cultures and her clinical findings. She was started on intravenous Solu-Medrol and was transitioned to oral prednisone on discharge based on her recent admission. Repeat laboratory tests during the patient’s subsequent admission demonstrated similarly elevated inflammatory markers and mildly elevated CK, supporting recurrent diabetic myonecrosis rather than an inflammatory process. During this hospital visit, she had a significant improvement in pain and edema with steroid therapy and was discharged.

Further workup, including a nuclear medicine scan of the body, re-demonstrated the increased uptake of the radiopharmaceutical in the right distal femur and proximal tibia, which was unchanged from the nuclear scan completed during her first admission, but now with soft tissue involvement of the right calf. The patient was monitored in the hospital for 6 days while on a steroid taper, and she had a marked improvement in her symptoms. Her glucose level had improved, and she was discharged on a slow prednisone taper, 20 mg for 7 days, followed by 10 mg for 7 days, then 5 mg for 7 days to prevent recurrence of her myonecrosis. During follow-up over the next 3 years, the patient remained recurrence-free. A summary of the patient’s clinical timeline, diagnostic findings, treatments, and outcomes is shown in **Figure 4**. Repeat laboratory tests during her second admission showed elevated ESR and CRP with a mildly elevated CK, consistent with her first episode.

## Discussion

Diabetic myonecrosis, or diabetic muscle infarction, is a rare but serious complication that primarily affects patients with a poorly controlled diabetes mellitus and is seen in patients with ESKD. Notably, diabetic myonecrosis occurs in both type 1 and type 2 diabetes mellitus, usually in patients with long-standing, poorly controlled disease, and management in either case remains centered on supportive care and the optimization of glycemic control. It manifests as a spontaneous ischemic necrosis of a single or a group of skeletal muscles that often manifests as acute pain (usually described as “out of proportion” to clinical findings) in addition to swelling and tenderness, primarily in the lower extremities. The pathophysiology is thought to involve a combination of microangiopathy with microvascular necrosis or capillary rarefaction, and it is seen in patients with significant atherosclerosis and/or hypercoagulable states. The final pathogenic characteristic is muscle ischemia and subsequent necrosis in the target muscles, with the resulting inflammation causing localized edema (1, 4, 5). The condition has been previously described in patients with poor glycemic control, as chronic hyperglycemia accelerates microvascular damage through endothelial dysfunction, increasing vascular permeability and rendering tissues more vulnerable to ischemia. While dyslipidemia can accelerate atherosclerosis and endothelial dysfunction (thereby worsening the overall vascular risk profile), an abnormal lipid panel has not been directly identified as an independent risk factor for diabetic myonecrosis beyond its contribution to the underlying vascular disease. In ESKD, it has been observed in patients with inadequate clearance due to missed dialysis treatments, although a clear association with calciphylaxis has not been demonstrated in this group (1, 3, 5–7). Of note is that our patient did not demonstrate any concerns for calciphylaxis, such as livedo reticularis or non-healing skin ulcers, to warrant alternative diagnoses. There was no evidence of vascular calcification involving cutaneous or subcutaneous tissues on imaging. Diabetics have a heightened pro-inflammatory and prothrombotic state, further impairing the muscle blood flow that is thought to promote muscle infarction (2, 8–11).

**Figure 3:**
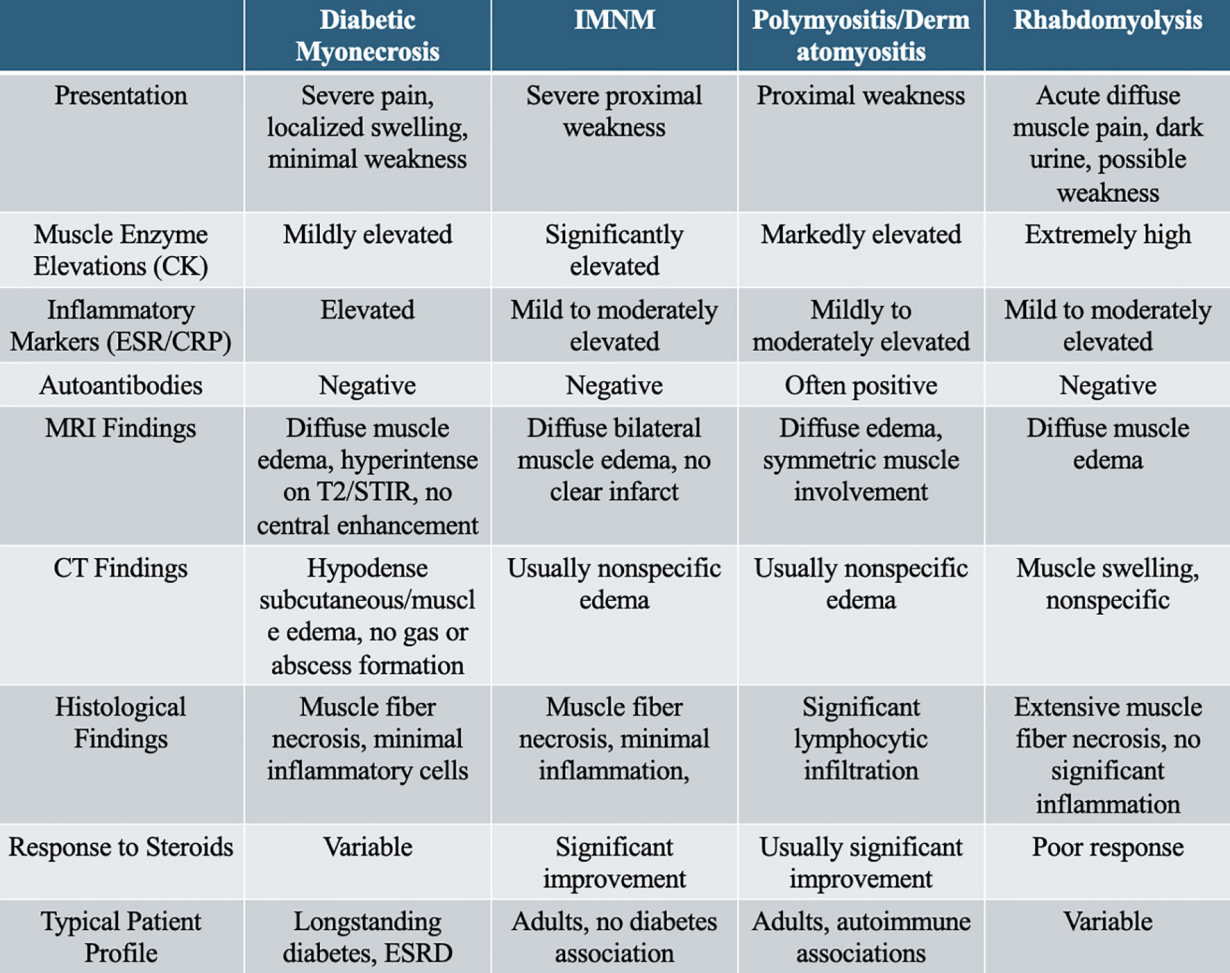
Key features between common pathologies. *CK*, creatine kinase; *ESR*, erythrocyte sedimentation rate; *CRP*, C-reactive protein; *IMNM*, immune-mediated necrotizing myopathy; *MRI*, magnetic resonance imaging; *T2/STIR*, T2-weighted/short tau inversion recovery.

This case depicts a patient with ESKD on hemodialysis who had a long-standing, poorly controlled diabetes mellitus, as evidenced by the frequent hyperglycemia and an HbA1c of 7.3%, notably her highest recorded value. Furthermore, HbA1c may underestimate the true glycemic status in dialysis patients due to anemia and erythropoiesis-stimulating agent use. She presented with a glucose level of 418 mg/dl, indicating sporadic poor glycemic control, which is not uncommon in those with type 1 diabetes, along with elevated inflammatory markers, but a fairly normal creatinine kinase. Most patients with myonecrosis are diagnosed either with cellulitis, abscess, muscle injury, or DVTs, and some have recurrence in their symptoms due to improper diagnosis (1, 2, 6, 8). Although high ESR and CRP levels could suggest an inflammatory myopathy, the absence of significantly elevated CK levels and a negative serology for the common myositis antibodies (e.g., Jo-1) support ischemic muscle injury (e.g., diabetic myonecrosis) rather than polymyositis or immune-mediated necrotizing myopathy (IMNM) (12, 13). Knee joint effusion is not a typical feature of diabetic myonecrosis, which affects the muscle rather than the joint; thus, the presence of a significant knee effusion would suggest an alternative or coexistent pathology (such as arthritis or infection) rather than being attributable to myonecrosis itself. The clinical presentation in these situations is critical. Key diagnostic indicators include the absence of fever or erythema (common in cellulitis), a negative duplex ultrasound for DVT, localized muscle tenderness without trauma history, and MRI demonstrating edema without abscess or infection. Additional laboratories that may point toward myonecrosis would be normal muscle enzyme levels, a negative autoimmune workup, and findings on imaging (e.g., CT or MRI) that indicate subcutaneous edema of the tissues without abscesses or subcutaneous infections. A history of absence of trauma to the affected muscles is critical, as was the case in this case. For clarity, **Figure 3** summarizes the key differentiating features between diabetic myonecrosis and other relevant myopathies and conditions.

**Figure 4:**
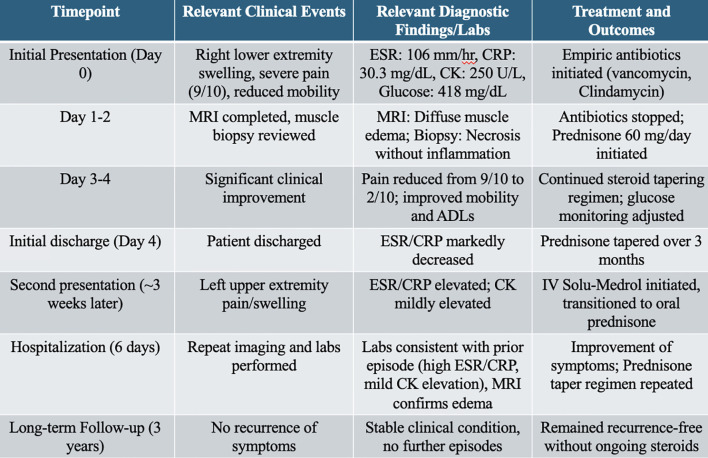
Relevant timeline of events.

Confusing the picture would be the elevation of the serum inflammatory (albeit nonspecific) biomarkers such as ESR and CRP. Although elevated ESR and CRP could suggest inflammatory myositis, the absence of markedly elevated CK levels and a negative serology (e.g., Jo-1 antibodies) strongly support diabetic myonecrosis rather than inflammatory conditions such as polymyositis or IMNM (12, 13). However, in this particular setting, the inflammation resulted from the sequelae of muscle injury and infarction, eventually leading to pain and edema. Similarly, the elevated alkaline phosphatase levels were likely secondary to bone mineral disease or osteodystrophy in a patient on hemodialysis for many years. IMNM, including seronegative variants, typically presents with severe proximal muscle weakness, significantly elevated CK levels, minimal systemic inflammatory markers, and marked steroid responsiveness, clearly distinguishing it from diabetic myonecrosis. Seronegative IMNM refers specifically to IMNM without detectable autoantibodies and typically manifests with severe proximal muscle weakness, markedly elevated CK levels, and minimal systemic inflammation. These features were notably absent in our patient. In contrast, diabetic myonecrosis, as observed in our patient, presents acutely with severe localized pain, mild CK elevations, pronounced systemic inflammation, and muscle biopsy findings of necrosis with minimal inflammatory infiltrates. These specific clinical, laboratory, and histologic findings strongly favored diabetic myonecrosis rather than IMNM in our patient’s case (12–15). This case depicts proper utilization of imaging with a high suspicion that could be useful in narrowing the diagnosis, as seen with the MRI findings that demonstrated muscle injury, edema, and a lack of infectious fluid collection such as abscess. Diagnosis ideally should include a muscle biopsy, which is the gold standard, although it was deferred since this was performed during her last admission at another hospital. A muscle biopsy can also distinguish between myonecrosis and myositis in a younger woman with ESKD who may have a concomitant autoimmune disease such as a mixed connective tissue disorder (1, 5).

The treatment of diabetic myonecrosis generally involves supportive care, including bed rest, analgesics, and optimization of glycemic control. In some cases, NSAIDs have been used in patients without significant kidney disease (1, 2, 5). In our patient, NSAIDs were contraindicated due to her prior allergy and a history of GI bleeding. Given the nature of her pain and her near immobility, as infectious causes were ruled out, a decision was made to treat her with steroids empirically. Despite limited literature supporting the use of corticosteroids in diabetic myonecrosis, the severe refractory pain, the marked inflammation, and the contraindications for NSAIDs justified this cautious empirical steroid trial (12, 13). Corticosteroids exert notable anti-inflammatory effects by reducing inflammatory cytokines such as interleukin 6 (IL-6) and tumor necrosis factor alpha (TNF-α), which are elevated during muscle ischemia and inflammation (2, 5, 7). Previous case reports of corticosteroid use in myonecrosis have not been promising. Our patient’s favorable response may be related to selective inflammation patterns, the recurrent phenotype, or the early therapeutic intervention, which differed from previously reported cases (16, 17). Our patient improved significantly within 48 h after the beginning of high-dose steroid therapy, primarily through mitigation of the inflammation and edema. While her glycemic control sporadically worsened due to corticosteroids, her recovery was significant. She was able to get off pain medications within 48 h and was able to ambulate and be discharged on day 4 with a steroid taper.

Steroids likely improve symptoms by minimizing the secondary inflammation without affecting the primary microangiopathy underlying myonecrosis. Long-term use of steroids in this population, particularly in diabetics, is not recommended as it is likely to worsen the glycemic control and its secondary complications. The risks of steroid use in patients with ESKD and diabetes include infection susceptibility, glycemic instability, fluid overload, osteoporosis, and other metabolic disturbances, necessitating careful consideration and monitoring. Furthermore, steroids accelerate microangiopathy in all populations. As previously mentioned, hyperglycemia exacerbates endothelial dysfunction and oxidative stress. It is also pro-inflammatory, all of which can heighten the risk of tissue ischemia, particularly in patients with preexisting vascular complications such as ESKD (8, 9). Even intermittent spikes in blood glucose can trigger significant vascular injury by promoting the formation of glycation end products (AGEs), contributing to endothelial dysfunction and vascular deterioration. This finding can explain the complex interplay between metabolic dysregulation and vascular/muscle health, emphasizing that glycemic variability can significantly impact the clinical outcomes (7, 11, 18, 19, 22).

However, in patients who have intractable pain or those who do not improve with conventional measures of supportive care, steroids may be an alternative that is used with supervision. This case posits numerous interesting questions. Currently, there is limited evidence to support their routine use, duration of therapy, and the target population, mainly due to significant gaps in medical knowledge about the etiopathogenesis of diabetic myonecrosis and the rare opportunity to initiate such a treatment.

Interestingly, this patient had recurrent episodes of myonecrosis in different muscle groups upon tapering steroid therapy. During her recurrence, she did respond to corticosteroids, but had a relapse as soon as the steroids were tapered, mimicking an inflammatory myositis. These episodes required weeks before any improvement, significantly affecting her mobility and functional status. Thus, whether these patients require a maintenance dose of steroids to prevent recurrence is an open question. It is also likely that racial and ethnic differences may pinpoint a genetic predisposition to the development of severe and recurrent myonecrosis in patients with ESKD (17).

Similarly, this steroid responsiveness also begs the question of whether such a population of patients may respond to steroid-sparing immunosuppression with a favorable side effect profile, e.g., calcineurin inhibitors or mycophenolate mofetil. It has to be noted that such interventions for a rare disease with uncertain pathophysiology are largely academic. Thus, any immunosuppression should be individualized and balanced with the risks of such therapies, particularly worsening glycemic control, cardiovascular disease, and infections (5, 20, 21, 22). The quantitative risks associated with steroids in diabetic patients with ESKD include significantly increased risks of hyperglycemia, infections (up to twofold higher), fluid retention exacerbating cardiac complications, and accelerated bone mineral loss, requiring careful evaluation against the clinical benefits.

## Conclusion

Diabetic myonecrosis is a rare but serious complication that is often difficult to diagnose and underscores the complex interplay between hyperglycemia, inflammation, and vascular compromise, particularly in patients with ESKD. Myonecrosis can be misdiagnosed as cellulitis or DVT, exposing affected patients to antibiotics or anticoagulants without any clinical improvement. A detailed workup, a high degree of suspicion, distinct clinical findings, and imaging such as MRI, along with muscle biopsy, can accurately diagnose this condition. Some patients with severe and recurrent symptoms may benefit from sporadic, short-term steroid therapy once an infection has been ruled out. Long-term use of steroids in those with recurrent symptoms has not been studied nor advocated due to the risk of worsening glycemic control in this population. To date, there is no evidence that any immunosuppressive medications (aside from the short-term corticosteroid therapy used in this case) provide added benefit in diabetic myonecrosis. Steroid-sparing immunosuppressants have only been speculated for refractory cases, but their efficacy remains unproven and they are not part of a standard management given their potential risks. Steroids likely work by mitigating the secondary inflammation and edema and can reduce the pain and the length of hospital stay in these patients. However, we emphasize that this represents a single case showing symptomatic improvement; broader recommendations regarding corticosteroid use in diabetic myonecrosis cannot be made without further studies and supporting evidence. This case emphasizes the importance of vigilance in managing glycemic variability and the need for further research to establish evidence-based treatment protocols for this elusive and challenging condition. The limitations of this case include its single-case nature, possible confounding factors, and the inability to definitively establish causality. Potential steroid risks, particularly significant in patients with ESKD, must be explicitly considered, which warrant further prospective research.

## Data Availability

The raw data supporting the conclusions of this article will be made available by the authors, without undue reservation.
